# Associations among circulating levels of follistatin-like 1, clinical parameters, and cardiovascular events in patients undergoing elective percutaneous coronary intervention with drug-eluting stents

**DOI:** 10.1371/journal.pone.0216297

**Published:** 2019-04-29

**Authors:** Tatsuro Aikawa, Kazunori Shimada, Katsumi Miyauchi, Tetsuro Miyazaki, Eiryu Sai, Shohei Ouchi, Tomoyasu Kadoguchi, Mitsuhiro Kunimoto, Yusuke Joki, Tomotaka Dohi, Shinya Okazaki, Kikuo Isoda, Koji Ohashi, Toyoaki Murohara, Noriyuki Ouchi, Hiroyuki Daida

**Affiliations:** 1 Department of Cardiovascular Medicine, Juntendo University Graduate School of Medicine, Tokyo, Japan; 2 Department of Molecular Medicine and Cardiology, Nagoya University Graduate School of Medicine, Nagoya, Japan; 3 Department of Cardiology, Nagoya University Graduate School of Medicine, Nagoya, Japan; International University of Health and Welfare, School of Medicine, JAPAN

## Abstract

**Objectives:**

Follistatin-like 1 (FSTL1) is a glycoprotein secreted by skeletal muscle cells and cardiac myocytes. Previous studies showed that serum FSTL1 concentrations were increased in acute coronary syndrome and chronic heart failure. The aim of this study was to assess the associations among plasma FSTL1 concentration, clinical parameters, and whether FSTL1 concentration could predict cardiovascular events in patients with elective percutaneous coronary intervention (PCI).

**Methods and results:**

A consecutive series of 410 patients who underwent elective PCI with drug-eluting stents (DES) were enrolled between August 2004 and December 2006 at Juntendo University hospital. We measured plasma FSTL1 levels prior to elective PCI and assessed the association among FSTL1 levels, clinical parameters, and occurrence of major adverse cardiac or cerebrovascular events (MACCE) defined as cardiac death, nonfatal myocardial infarction, unstable angina, stroke, and hospitalization for heart failure. FSTL1 concentration was positively correlated with high-sensitivity C-reactive protein (hsCRP), serum creatinine, and N-terminal pro b-type natriuretic peptide (all *P* < 0.01). After excluding patients with creatinine clearance < 60 mL/min and hsCRP ≥ 0.2 mg/dL, the remaining 214 were followed for a median of 5.1 years. Twenty (9.3%) patients experienced MACCE. Receiver operating characteristics curve analysis estimated an FSTL1 cutoff of 41.1 ng/mL to predict MACCE occurrence. Kaplan–Meier analysis found a higher MACCE rate in patients with high (≥ 41.1 ng/mL) than with low (< 41.1 ng/mL) FSTL1 (*P* < 0.01). Multivariate Cox hazard analysis found that high FSTL1 was an independent predictor of MACCE (hazard ratio 4.54, 95% confidence interval: 1.45–20.07, *P* < 0.01).

**Conclusion:**

High plasma FSTL1 may be a predictor of cardiovascular events in patients who underwent elective PCI with DES, especially with preserved renal function and low hsCRP.

## Introduction

Follistatin-like 1 (FSTL1) is a glycoprotein, also known as transforming growth factor (TGF)-β1–stimulated clone 36 (TSC-36) [1, 2], synthesized and secreted by skeletal muscle cells and cardiomyocytes. It was shown that Fstl1 is a cardiokine upregulated by various heart stresses, including cardiac ischemia/reperfusion injury, pressure overload, and myocardial infarction [3–5]. Wei et al. showed that Fstl1 is expressed in epicardial cells surrounding the myocardium, and that Fstl1 can induce cardiomyocyte proliferation of the normal adult mouse heart. Furthermore, Fstl1 is disappears from epicardial cells in the mouse heart with myocardial infarction [6]. Previous clinical studies showed that serum FSTL1 concentrations were increased in patients with acute coronary syndrome (ACS) [7] and chronic systolic heart failure (HF) [8]. Widera et al. found that FSTL1 concentrations were increased in ACS and associated with all-cause mortality [7]. El-Armouche et al. found that elevated serum FSTL1 in patients with HF was associated with left ventricular hypertrophy (LVH) [8]. These results suggested that FSTL1 may be a useful marker for evaluation of cardiovascular disease. Hayakawa et al. demonstrated that plasma FSTL1 levels were correlated with hsCRP and reactive oxidative metabolites (ROMs) in healthy Japanese male subjects [9], and their results indicate that FSTL1 may be a biomarker for inflammatory and oxidative stress responses. However, whether FSTL1 concentration could predict cardiovascular events in patients with coronary artery disease (CAD) have not fully investigated. The aim of this study is to clarify the association of FSTL1 concentration and clinical parameters, and the occurrence of major adverse cardiac or cerebrovascular events (MACCE) in patients with elective percutaneous coronary intervention (PCI).

## Materials and methods

### Study subjects

A consecutive series of 410 patients who underwent elective PCI with first-generation drug-eluting stents (DES) were enrolled at Juntendo University Hospital between August 2004 and December 2006. Patients with ACS, malignant disease, apparent inflammatory disease, active liver disease and/or liver cirrhosis, or acute decompensated heart failure (ADHF) were excluded. The study protocol conforms to the ethical guidelines of the Declaration of Helsinki. The study was approved by the ethics committee of Juntendo University Hospital, and written informed consent was obtained from all patients before participation.

### Clinical variables

We measured plasma FSTL1 levels prior to elective PCI on the same day. We collected blood samples in the operating room just before the elective PCI was performed, and after centrifugation, the plasma samples were stored at − 80°C. We assessed the association between FSTL1 levels and clinical parameters.

Demographic data, coronary risk factors, and medication were collected from our institutional database. Blood samples were collected early in the morning after fasting overnight. Plasma total cholesterol (TC) and creatinine were assayed by standard enzymatic methods. Triglycerides (TG) were assayed by visible absorption spectrometry, high-density lipoprotein cholesterol (HDL-C) levels by the direct method, and low-density lipoprotein cholesterol (LDL-C) was determined the Friedewald formula [10]. N-terminal pro B-type natriuretic peptide (NT-proBNP) was determined by electrochemiluminescence–immunoassay, hemoglobin A1c (HbA1c) was assayed by high-performance liquid chromatography, and serum hsCRP was measured using a validated, highly sensitive immunoassay and particle-enhanced immunonephelometry (Dade Behring Holding GmbH, Liederbach, Germany). Plasma FSTL1 was determined with an enzyme-linked immunosorbent assay kit (USCN Life Science, Inc., Houston, USA) in accordance with the manufacturer’s instructions. Briefly, the microplates were pre-coated with an antibody specific to FSTL1, and standards or samples were incubated in the wells with a biotin-conjugated antibody specific to FSTL1. Avidin conjugated to horseradish peroxidase (HRP) was added to each well, and the TMB substrate solution was added. Finally, sulfuric acid solution was added to terminate the enzyme–substrate reaction. The color change was measured spectrophotometrically at a wavelength of 450 nm. Standards and samples were measured in duplicate and averaged.

Blood pressure (BP) was measured on admission with a mercury sphygmomanometer. Hypertension was defined as a systolic BP ≥ 140 mmHg, diastolic BP ≥ 90 mmHg, or the current use of antihypertensive medications. Dyslipidemia was defined as LDL-C ≥ 140 mg/dL, HDL-C < 40 mg/dL, TGs ≥ 150 mg/dL, or current treatment with statins and/or other lipid-lowering medications. Diabetes mellitus was defined as either HbA1c ≥ 6.5% or current use of insulin or oral hypoglycemic drugs. Chronic kidney disease (CKD) required a creatinine clearance (CCr < 60 mL/min) and was calculated with the Cockroft-Gault equation as [(140 − age) × weight (kg) / 72 × serum creatine (mg/dL)]. For women, the result was multiplied by 0.85 [11–13].

### Angiography

Coronary angiography was performed on all patients at baseline. Clinically significant stenosis was measured by the number of stenotic vessels recorded as 1-, 2-, or 3-vessel disease (> 75% narrowing) or as stenosis of the left main coronary artery (> 50% narrowing). All procedural decisions, including device selection and adjunctive pharmacotherapy, were made at the discretion of each PCI operator. Intravenous unfractionated heparin and intracoronary nitroglycerin were administered before PCI procedures. Angiographic optimization of stent implantation was performed using high-pressure dilatation to achieve an acceptable angiographic result. Intravascular ultrasound was performed at the operator’s discretion. Procedural success was defined as residual stenosis of < 20% without major complications. Dual antiplatelet therapy with 100 mg aspirin and 200 mg ticlopidine or 75 mg clopidogrel was prescribed for all patients implanted with a DES for at least 1 year.

### Follow-up

The study participants were followed at our hospital or affiliated hospitals until March 2011. The outcomes of patients who died were retrieved from their medical records. Clinical outcomes were collected from the medical records of patients who died. MACCE is defined as cardiac death, ACS, stroke, and hospitalization for ADHF. ACS was defined as unstable angina pectoris (UAP), non-ST segment elevation myocardial infarction (NSTEMI), or ST segment elevation myocardial infarction (STEMI). Myocardial infarction was defined as a ≥ 2-fold increase in serum creatine kinase and troponin T positivity. UAP was diagnosed as angina at rest or in an accelerating pattern with negative cardiac biomarkers with or without electrocardiogram (ECG) changes indicative of myocardial ischemia. ADHF was defined by the Framingham study criteria [14]. Cardiac deaths were those caused by HF or MI or were recorded as sudden death. Multivessel coronary disease was defined as presence of coronary artery disease of two or more vessels with visually assessed stenosis of > 75% following the American Heart Association Classification [15, 16].

### Statistical analysis

Continuous variables were expressed as means ± standard deviation (SD) or medians with 25th and 75th percentiles. Categorical variables were reported as percentages. Between-group differences were compared with the unpaired Student’s *t*-test, chi-square test, Fisher’s exact test, or the Mann–Whitney–Wilcoxon rank-sum test, as appropriate. Correlations of two variables were determined by simple linear regression analysis. Spearman correlations were calculated if variables were not normally distributed. Plasma FSTL1, hsCRP, and NT-proBNP concentrations were transformed to their natural logarithm (log) values for the regression analysis because of their skewed distributions. The Kaplan–Meier method was used to estimate the cumulative probability of outcomes, and between-group differences were compared with the log-rank test. The cutoff FSTL1 value was estimated by receiver operating characteristics (ROC) curve analysis. Univariate and multivariate Cox hazard analyses were performed to identify predictors of the primary endpoint, and hazard ratios (HRs) with their 95% confidence intervals (CIs) were calculated. Multivariate Cox hazard analysis was used to identify variables independently associated with MACCE among those with a *P*-value ≤ 0.05 in univariate analysis (TG, HbA1c, NT-proBNP, diuretic, FSTL1 ≥ 41.1 ng/mL), age, sex, and body mass index (BMI). Statistical analysis was performed with JMP 12 software for Windows (SAS Institute, Cary, NC, USA.). Statistical significance was defined as a *P*-value ≤ 0.05.

## Results

### Patient characteristics and outcomes

The characteristics of the 410 study participants are shown in Table 1. The median FSTL1 was 49.5 (33.1–81.8) ng/mL, and the range was 6.0–567.4 ng/mL. The correlates of Log FSTL1 after univariate linear regression analyses are shown in Table 2. Log FSTL1 was negatively correlated with CCr and positively correlated with Log hsCRP and Log NT-proBNP (all *P* < 0.0001). Plasma FSTL1 was significantly higher in patients with CCr < 60 mL/min than those with CCr ≥ 60 mL/min (*P* < 0.0001, S1 Fig). Sixty-three of the 410 patients (15.3%) experienced a MACCE. Ten (2.4%) cardiac deaths occurred, 28 (6.8%) patients experienced ACS, 19 (4.6%) experienced ADHF, and 6 (1.5%) experienced stroke. Plasma FSTL1 was significantly higher in patients with than in those without MACCE (80.8 ± 70.1 vs. 66.9 ± 61.2 ng/mL, *P* = 0.019). However, after adjusting for confounding factors, FSTL1 was not significantly associated with the occurrence of MACCE in the overall study population.

**Table 1:** Patient characteristics of the study population.

Parameters	Subjects (n = 410)
Age, (years)	65.6 ± 8.7
Male, n (%)	352 (85.9)
Body mass index (kg/m^2^)	24.2 ± 3.2
Hypertension, n (%)	308 (75.1)
Dyslipidemia, n (%)	298 (72.7)
Diabetes mellitus, n (%)	218 (53.2)
Current smoking, n (%)	100 (24.4)
ESKD (CCr < 15/mL/min), n (%)	21 (5.1)
Previous myocardial infarction, n (%)	121 (29.6)
Previous PCI, n (%)	166 (40.6)
Previous CABG, n (%)	53 (13.0)
No. of diseased vessels	
One, n (%)	133 (32.5)
Two, n (%)	149 (36.3)
Three, n (%)	128 (31.2)
Stenosis of LMT, n (%)	11 (2.7)
Multi vessel disease, n (%)	277 (67.6)
Target lesion	
LAD, n (%)	176 (42.9)
LCX, n (%)	99 (24.2)
RCA, n (%)	124 (30.2)
LMT, n (%)	11 (2.7)
Bifurcation lesion, n (%)	33 (8.1)
Number of stents	
One, n (%)	322 (78.5)
Two, n (%)	74 (18.1)
Three, n (%)	13 (3.2)
Four, n (%)	1 (0.2)
Total cholesterol (mg/dL)	175 ± 33
LDL-cholesterol (mg/dL)	107 ± 30
Triglyceride (mg/dL)	129 ± 65
HDL-cholesterol (mg/dL)	44 ± 12
Fasting blood glucose (mg/dL)	112.3 ± 38.5
Hemoglobin A1c (%)	6.6 ± 1.3
Creatinine (mg/dL)	1.3 ± 2.0
Creatinine clearance (mL/min)	74.8 ± 28.6
High-sensitivity CRP (mg/dL)	0.080 (0.037, 0.21)
NT-proBNP (pg/mL)	136.9 (62.8, 436.8)
Follistatin-like 1 (ng/mL)	69.1 ± 62.7 49.5 (33.1, 81.8)
Left ventricular ejection fraction (%)	60.8 ± 11.5
Medications	
Antiplatelet, n (%)	410 (100)
Calcium channel blocker, n (%)	165 (40.2)
β-blocker, n (%)	236 (57.6)
ACE inhibitor or ARB, n (%)	202 (49.3)
Diuretic, n (%)	35 (8.5)
Statin, n (%)	231 (56.3)

Values are means ± SD, or medians (25th–75th percentile); ESKD, end stage kidney disease; CCr, creatinine clearance; PCI, percutaneous coronary intervention; CABG, coronary artery bypass grafting; LMT, left main trunk; LAD, left anterior descending artery; LCX, left circumflex; RCA, right coronary artery; LDL, low-density lipoprotein; HDL, high-density lipoprotein; CRP, C-reactive protein; NT-proBNP, N-terminal pro B-type natriuretic peptide; ACE, angiotensin converting enzyme; ARB, angiotensin II receptor blocker.

**Table 2:** Correlations of log FSTL1 and clinical characteristics.

	*r*	*P*
Age	0.10	< 0.05
Body mass index	−0.04	0.46
Creatinine	0.27	< 0.0001
Creatinine clearance	−0.23	< 0.0001
Hemoglobin A1c	−0.08	0.09
Total cholesterol	−0.06	0.23
LDL-cholesterol	−0.02	0.74
Triglyceride	−0.04	0.42
HDL-cholesterol	−0.18	< 0.001
Log High-sensitivity CRP	0.26	< 0.0001
Log NT-proBNP	0.29	< 0.0001
LVEF	−0.08	0.15

Single linear regression analysis association with Log FSTL1. FSTL1, follistatin-like 1; LDL, low-density lipoprotein; HDL, high-density lipoprotein; CRP, C-reactive protein; NT-proBNP, N-terminal pro-B-type natriuretic peptide; LVEF, left ventricular ejection fraction.

**Fig 1:**
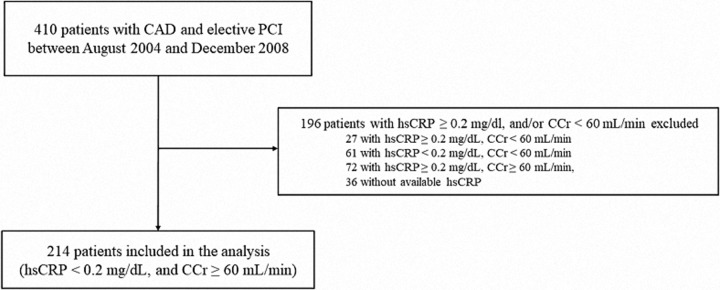
Study flow chart showing the disposition and outcome of 214 patients. Those with CCr < 60 mL/min and hsCRP ≥ 0.2 mg/dL were excluded. During a median 5.1-year follow-up, 20 patients experienced MACCE. CAD, coronary artery disease; PCI, percutaneous coronary intervention; hsCRP, high-sensitivity C-reactive protein; CCr, creatinine clearance; MACCE, major adverse cardiac or cerebrovascular events; ACS, acute coronary syndrome; CHF, congestive heart failure.

**Fig 2:**
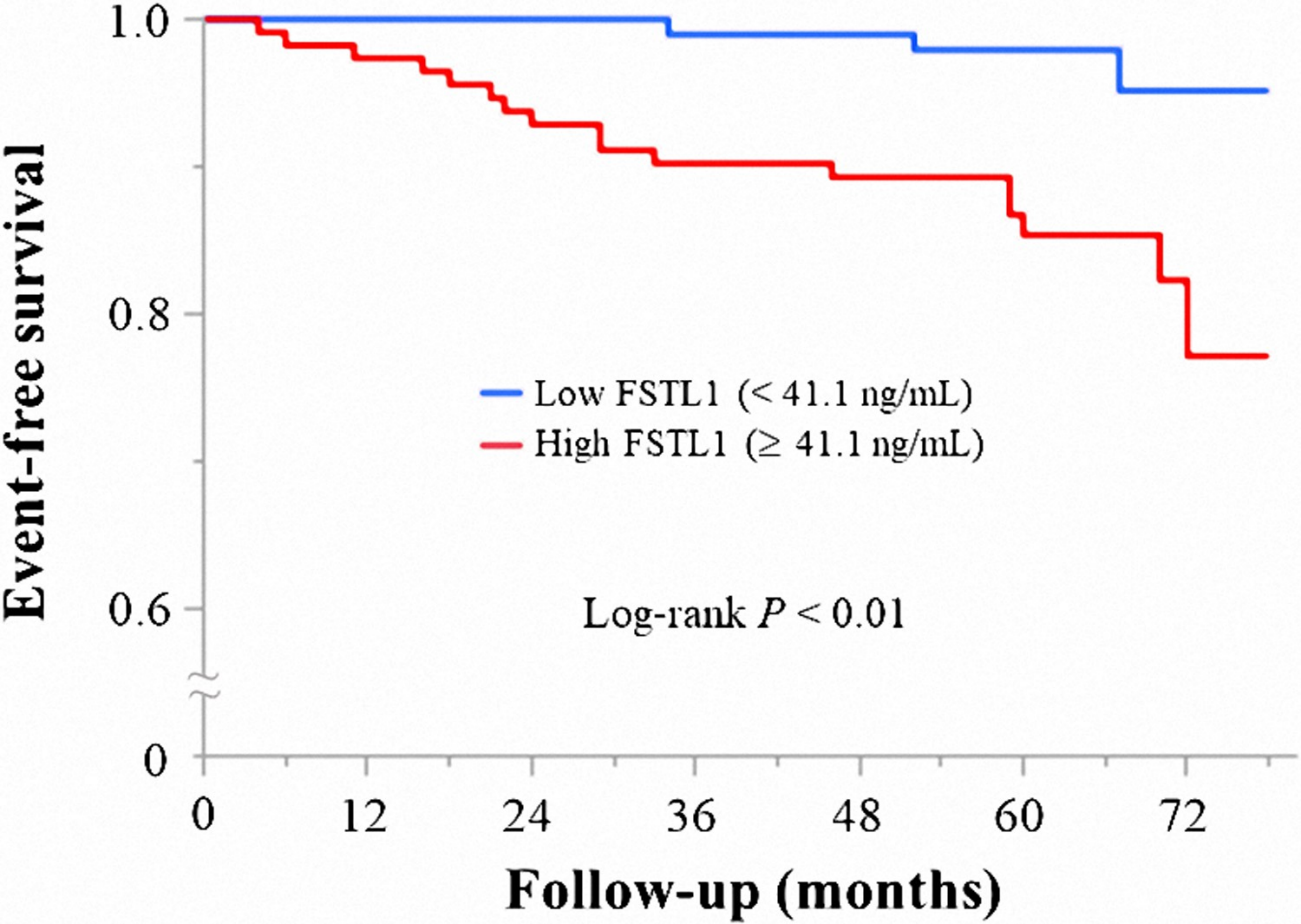
Event-free survival for MACCE. Kaplan–Meier analysis revealed that the MACCE rate was significantly higher in patients with FSTL1 (≥ 41.1 ng/mL) than in those FSTL1 < 41.1 ng/mL (*P* < 0.01). FSTL1, follistatin-like 1; MACCE, major adverse cardiac or cerebrovascular events.

In line with previous reports, FSTL1 was elevated in patients with CKD and/or high levels of systemic inflammation [7, 8]. Therefore, we followed 214 patients after excluding patients with CCr < 60 mL/min and hsCRP ≥ 0.2 mg/dL [17, 18]. During the median 5.1 years of follow-up, 20 patients experienced MACCE. There were 3 (1.4%) cardiac deaths, 12 patients (5.6%) experienced ACS, and 5 (2.3%) had HF (Fig 1). The characteristics of the 214 patients are shown in Table 3. The median FSTL1 concentration was 43.2 (28.7–70.3) ng/mL, and the range was 8.6–466.0 ng/mL. FSTL1 was significantly higher in patients with than without MACCE (86.0 ± 93.8 vs. 54.0 ± 40.5 ng/mL, *P* < 0.05). Firstly, we divided the 214 patients into two groups based on the median FSTL1 levels (43.2 ng/mL), and Kaplan–Meier analysis showed a higher MACCE rate in patients with high (≥ 43.2 ng/mL) than in those with low (< 43.2 ng/mL) FSTL1 (*P* < 0.05, S2 Fig) level. Multivariate Cox hazard analyses demonstrated that high FSTL1 level was an independent predictor of MACCE (hazard ratio 2.75, 95% confidence interval: 1.01–8.71, *P* < 0.05) (S1 Table). Then, patients were divided into three groups as per the FSTL1 level (< 34.0 ng/mL, 34.0 ng/mL to 62.3 ng/mL, and > 62.3 ng/mL), and the Kaplan–Meier analysis showed the same trend; however, no significant difference was noted among the three groups (*P* = 0.063, S3 Fig). Next, we performed ROC analysis to determine the optimal cutoff value for FSTL1 level. ROC curve analysis estimated that an FSTL1 cutoff value of 41.1 ng/mL had a sensitivity of 85% and specificity of 49% to predict the occurrence of MACCE (area under the curve, 0.68; *P* < 0.05, S4 Fig). When the patients were stratified by FSTL1 concentration, Kaplan–Meier analysis found that those with high (≥ 41.1 ng/mL) FSTL1 had a significantly higher rate of MACCE occurrence within the follow-up period than those with low concentrations (< 41.1 ng/mL, *P* < 0.01; Fig 2). Univariate Cox hazard analysis showed that TG (HR 1.01, 95% CI: 1.00–1.01, *P* < 0.05), HbA1c (HR 1.28, 95% CI: 1.00–1.57, *P* < 0.05), NT-proBNP (HR 1.001, 95% CI: 1.000–1.002, *P* < 0.01), diuretic use (HR 6.90, 95% CI: 2.44–17.22, *P* < 0.001), and FSTL1 ≥ 41.1 ng/mL (HR 5.43, 95% CI: 1.82–23.25, *P* < 0.01) were associated with MACCE (Table 4). Multivariate Cox hazard analysis showed that FSTL1 ≥ 41.1 ng/mL was an independent predictor of MACCE (HR 4.54, 95% CI: 1.45–20.07, *P* < 0.01; Table 4).

**Table 3:** Clinical characteristics of patients with preserved renal function, and low hsCRP.

	All patients N = 214	No MACCE N = 194	MACCE N = 20	*P*
Age, (years)	63.8 ± 7.9	64.0 ± 7.9	61.6 ± 8.0	0.13
Male, n (%)	189 (88.3)	173 (89.2)	16 (80.0)	0.26
Body mass index (kg/m^2^)	24.6 ± 2.9	24.5 ± 2.8	25.2 ± 3.3	0.45
Hypertension, n (%)	164 (76.6)	147 (75.8)	17 (85.0)	0.42
Dyslipidemia, n (%)	157 (73.4)	141 (72.7)	16 (80.0)	0.60
Diabetes mellitus, n (%)	115 (53.7)	101 (52.1)	14 (70.0)	0.15
Current smoking, n (%)	49 (22.9)	42 (21.7)	7 (35.0)	0.17
Previous MI, n (%)	63 (29.4)	55 (28.4)	8 (40.0)	0.30
Previous PCI, n (%)	89 (41.6)	77 (39.7)	12 (60.0)	0.09
Previous CABG, n (%)	26 (12.2)	24 (12.4)	2 (10.0)	0.99
No. of diseased vessels				0.80
One, n (%)	78 (36.5)	72 (37.1)	6 (30.0)	
Two, n (%)	71 (33.1)	64 (33.0)	7 (35.0)	
Three, n (%)	65 (30.4)	58 (29.9)	7 (35.0)	
Stenosis of LMT, n (%)	5 (2.3)	4 (2.1)	1 (5.0)	0.40
Multi vessel disease, n (%)	136 (63.6)	122 (62.9)	14 (70.0)	0.63
Target lesion				0.71
LAD, n (%)	91 (42.5)	82 (42.2)	9 (45.0)	
LCX, n (%)	57 (26.7)	51 (26.3)	6 (30.0)	
RCA, n (%)	61 (28.5)	57 (29.4)	4 (20.0)	
LMT, n (%)	5 (2.3)	4 (2.1)	1 (5.0)	
Bifurcation lesion, n (%)	16 (7.5)	15 (7.7)	1 (5.0)	0.99
Number of stents				0.058
One, n (%)	176 (82.3)	161 (83.0)	15 (75.0)	
Two, n (%)	33 (15.4)	30 (15.5)	3 (15.0)	
Three, n (%)	5 (2.3)	3 (1.5)	2 (10.0)	
Total cholesterol (mg/dL)	173 ± 31	173 ± 31	176 ± 31	0.58
LDL-cholesterol (mg/dL)	106 ± 28	106 ± 28	106 ± 28	0.90
Triglyceride (mg/dL)	128 ± 61	125 ± 57	160 ± 85	< 0.05
HDL-cholesterol (mg/dL)	44 ± 11	45 ± 11	40 ± 10	0.065
Hemoglobin A1c (%)	6.6 ± 1.4	6.5 ± 1.4	7.3 ± 1.8	< 0.05
Creatinine (mg/dL)	0.83 ± 0.17	0.83 ± 0.17	0.82 ± 0.17	0.70
CCr (mL/min)	85.6 ± 21.7	85.0 ± 20.5	91.7 ± 31.1	0.48
High-sensitivity CRP (mg/dL)	0.052 (0.028, 0.092)	0.052 (0.026, 0.092)	0.061 (0.032, 0.11)	0.31
NT-proBNP (pg/mL)	90 (45, 200)	83 (44, 180)	189 (80, 794)	< 0.01
FSTL1 (ng/mL)	57.0 ± 48.5 43.2 (28.7, 70.3)	54.0 ± 40.5 41.3 (26.7, 68.2)	86.0 ± 93.8 58.9 (43.0, 100.7)	< 0.01
LVEF (%)	62.3 ± 10.3	62.8 ± 10.1	57.2 ± 11.6	< 0.05
Medications				
Antiplatelet, n (%)	214 (100)	194 (100)	20 (100)	1.0
CCB, n (%)	86 (40.2)	78 (40.2)	8 (40.0)	1.0
β-blocker, n (%)	128 (59.8)	118 (60.8)	10 (50.0)	0.35
ACE inhibitor or ARB, n (%)	110 (51.4)	97 (50.0)	13 (51.4)	0.24
Diuretic, n (%)	13 (6.1)	7 (3.6)	6 (30.0)	< 0.001
Statin, n (%)	127 (59.3)	113 (58.3)	14 (70.0)	0.34

Values are means ± SD, or medians (25th–75th percentile), MACCE, major adverse cardiac or cerebrovascular events; MI, myocardial infarction; PCI, percutaneous coronary intervention; CABG, coronary artery bypass grafting; LMT, left main trunk; LAD, left anterior descending artery; LCX, left circumflex; RCA, right coronary artery; LDL, low-density lipoprotein; HDL, high-density lipoprotein; CCr, creatinine clearance; CRP, C-reactive protein; NT-proBNP, N-terminal pro-B-type natriuretic peptide; FSTL1, follistatin-like 1; LVEF, left ventricular ejection fraction; CCB, calcium channel blocker; ACE, angiotensin converting enzyme; ARB, angiotensin II receptor blocker.

**Table 4:** Multivariate cox proportional hazard models for MACCE.

	Univariate			Multivariate		
	HR	95% CI	*P*	HR	95% CI	*P*
Age, 1year increase	0.96	0.91–1.02	0.16	0.97	0.91–1.03	0.28
Gender (Male)	0.50	0.18–1.75	0.24	0.37	0.11–1.50	0.12
Body mass index, 1kg/m^2^ increase	1.10	0.94–1.25	0.20	1.04	0.88–1.20	0.65
Triglyceride, 1 mg/dL increase	1.01	1.00–1.01	<0.05	1.01	0.99–1.01	0.08
Hemoglobin A1c, 1% increase	1.28	1.00–1.57	<0.05	1.03	0.75–1.37	0.82
NT-pro BNP, 1 pg/mL increase	1.001	1.000–1.002	<0.01	1.001	0.999–1.002	0.41
Diuretic usage	6.90	2.44–17.22	<0.001	2.98	0.51–13.38	0.21
FSTL1 ≥ 41.1 ng/mL	5.43	1.82–23.25	<0.01	4.54	1.45–20.07	<0.01

MACCE, major adverse cardiac or cerebrovascular events; HR, hazard ratio; CI, confidence interval; NT-proBNP, N terminal pro brain natriuretic peptide; FSTL-1, follistatin-like 1.

## Discussion

The present study demonstrated that plasma FSTL1 was significantly increased in CKD patients and was positively correlated with hsCRP in patients with CAD. After excluding patients with CKD and relatively high systemic inflammation, those with FSTL1 ≥ 41.1 ng/mL had a significantly higher rate of MACCE occurrence than those with FSTL1 < 41.1 ng/mL. High plasma FSTL1 predicted cardiovascular events in patients who underwent elective PCI with first-generation DES, especially with preserved renal function and relatively low-grade systemic inflammation.

Although the effects of Fstl1 in cardiovascular and inflammatory disorders have been previously demonstrated in basic studies including animal models, clinical function and evidence of FSTL1 are limited. Widera et al. reported that serum FSTL1 levels were significantly higher and related to all-cause mortality in patients with ACS [7], and high FSTL1 levels were associated with a higher risk of cardiovascular death in patients with ACS [19]. They measured the circulating concentrations of FSTL1 and growth differentiation factor 15 (GDF15) in 1369 patients with ACS. GDF15 is a stress-responsive cytokine induced after injury in the heart and vasculature. They identified FSTL1 as an activator of GDF 15 gene expression in vitro and vivo. In addition, they showed the circulating concentrations of FSTL1 to be independently related to GDF15 and to cardiovascular mortality in ACS. FSTL1 and GDF15 may reflect overlapping disease pathways in ACS. FSTL1 remained associated with cardiovascular death after adjustment for clinical, angiographic, and biochemical variables. The increase in serum FSTL1 was reported to be similar in patients with UAP, NSTEMI, and STEMI, indicating that FSTL1 may not be related to the extent of myocardial necrosis during an episode of ACS [7].

In this study, plasma FSTL1 ≥ 41.1 ng/mL was associated with MACCE including those patients admitted with ADHF. El-Armouche et al. reported that serum FSTL1 levels were significantly increased in chronic HF patients with reduced systolic function and was associated with LVH and NT-proBNP [8]. Lara-Pezzi et al. reported that FSTL1 expression was elevated in cardiac myocytes and endothelium in patients with HF and returned to normal levels after recovery of myocardial function who received combined left ventricular assist device and pharmacological therapy [20]. The evidence may indicate that the FSTL1 levels existing before HF treatment are associated with long-term outcome. A report that plasma FSTL1 levels were correlated with hsCRP and ROMs in healthy Japanese male subjects suggests that FSTL1 may be a biomarker of metabolic dysfunction and cardiovascular disease [9]. Hayakawa et al. also reported that plasma Fstl1 levels increased in mice after renal injury, which was accompanied by an increase in cardiac Fstl1 protein levels [21]. Plasma hsCRP level predicts the risk of MI and ischemic stroke [22], and preprocedural hsCRP level is an independent predictor of cardiovascular events after PCI [23–25]. We previously reported that preprocedural hsCRP in patients with CKD was associated with poor outcomes after PCI with first-generation DES [26]. We also reported that elevated preprocedural hsCRP was associated with long-term clinical outcome in patients after PCI [27]. In this study, plasma FSTL1 was significantly associated with CCr and hsCRP in patients with CAD, but plasma FSTL1 in patients with and without MACCE were not significantly different when patients with renal dysfunction and/or high-grade systemic inflammation were included. After excluding patients with CCr < 60 mL/min and hsCRP ≥ 0.2 mg/dL [17, 18, 28, 29], plasma FSTL1 levels were significantly higher in patients with MACCE than in those who did not experience MACCE and was significantly associated with the occurrence of cardiovascular events. FSTL1 level may predict cardiovascular events, especially in patients with preserved renal function and relatively low-grade inflammatory state.

The function of FSTL1 in the cardiovascular system seems to be poorly understood in patients with cardiovascular disease. It is still unknown whether a high plasma FSTL1 concentration simply reflects underlying disease processes or has a maladaptive and/or compensatory role in modulating the pathogenesis of cardiac dysfunction in patients with cardiovascular disease. Fstl1 is secreted by skeletal muscle cells and cardiac myocytes in response to hypertrophy and ischemic injury [30, 31]. Fstl1 stimulates endothelial cell migration and survival via activation of Akt signaling [32]. Fstl1 also reduces cardiomyocyte apoptosis through the activation of Akt or AMP-activated protein kinase (AMPK) signaling [3, 4]. Thus, it is likely that Fstl1 serves as a survival factor for both cardiac myocytes and endothelial cells. Furthermore Miyabe et al. showed the role of Fstl1 in proliferative vascular disease. Fstl1 reduces neointimal hyperplasia in injured arteries in vivo and inhibits the proliferation and migration of vascular smooth muscle cells in vitro via activation of AMPK [33]. Further study is needed to clarify the mechanism of FSTL1 in the cardiovascular system.

### Limitations

This study had several limitations. First, the study was conducted in a single institution and the study population was relatively small. Studies with a larger sample size, racial diversity, and multicenter will be required to better define the role of plasma FSTL1 as a prognostic biomarker for patients with stable CAD. Second, we measured plasma FSTL1 level only in patients with CAD. Further studies are needed to assess the role of FSTL1 on the initiation of CAD. Third, we measured plasma FSTL1 levels just before elective PCI. We did not assess the changes in plasma FSTL1 levels between the baseline and follow-up. Fourth, the sample size for women was relatively small. However, it tended to be similar to the proportion of men and women reported in a previous study [16]. Moreover, we performed sex-stratified sensitivity analysis in 189 patients after excluding women. The results of the ROC curve analysis and Kaplan–Meier analysis showed the same trends (data not shown). Fifth, compared to the number of patients with dyslipidemia, the prevalence of statin use was relatively low. We thought that patients’ historical background may have influenced the administration rate of statin. Sixth, we performed stepwise regression analysis, including FSTL1, age, sex, hypertension, dyslipidemia, diabetes, current smoking, and NT-proBNP in the 214 patients. As continuous variables, FSTL1 level showed a trend but was not a significant independent predictor of MACCE (data not shown). As described above, FSTL1 levels were associated with LVH and NT-proBNP [8]. In addition, the sample size of the present study was relatively small. Therefore, larger sample-sized prospective studies are required to assess the relationships between FSTL1 and NT-proBNP levels. Finally, 70% of the patients with MACCE had diabetes that involves a pro-inflammatory state. The results of the JUPITER trial suggested that high levels of hsCRP (> 0.2 mg/dL) represent a chronic inflammatory state and a high risk of cardiovascular events [17]. Diabetes also represents a pro-inflammatory state. Therefore, diabetic patients with low levels of hsCRP (< 0.2 mg/dL) may also be in a low-grade inflammatory state.

## Conclusions

A high plasma FSTL1 concentration may be a predictor of cardiovascular events only in patients with CAD who underwent elective PCI with DES and with preserved renal function and a relatively low-grade inflammatory state. Future studies are needed to validate its usefulness as a biomarker in patients with CAD.

## Supporting information

S1 Fig Plasma FSTL1 in Patients with CCr ≥ 60 mL/min and < 60 mL/min. Patients with CCr < 60 mL/min had higher mean plasma FSTL1 than those with CCr ≥ 60 mL/min. FSTL1 levels were expressed as medians with the 25th and 75th percentiles. FSTL1, follistatin-like 1; CCr: creatinine clearance.
(TIF)

S2 Fig Event-free survival for MACCE in two groups based on the median FSTL1 levels (43.2 ng/mL). Kaplan–Meier analysis revealed that the MACCE rate was significantly higher in patients with FSTL1 (≥ 43.2 ng/mL) than in those FSTL1 < 43.2 ng/mL (*P* < 0.05). FSTL1, follistatin-like 1; MACCE, major adverse cardiac or cerebrovascular events.
(TIF)

S3 Fig Event-free survival for MACCE in three groups as per the FSTL1 level (< 34.0 ng/mL, 34.0 ng/mL to 62.3 ng/mL, and > 62.3 ng/mL). Kaplan–Meier analysis showed a trend but not a significant difference was noted among the three groups based on the tertiles FSTL1 levels. FSTL1, follistatin-like 1; MACCE, major adverse cardiac or cerebrovascular events.
(TIF)

S4 Fig Cutoff FSTL1 value for prediction of MACCE. Receiver operating characteristics curve analysis estimated that an FSTL1 concentration of 41.1 ng/mL (area under the curve 0.68) had a sensitivity of 85% and specificity 49% for predicting MACCE (*P* < 0.05). FSTL1, follistatin-like 1; MACCE, major adverse cardiac or cerebrovascular events.
(TIF)

S1 Table Multivariate cox proportional hazard models for MACCE. MACCE, major adverse cardiac or cerebrovascular events; HR, hazard ratio; CI, confidence interval; NT-proBNP, N terminal pro brain natriuretic peptide; FSTL-1, follistatin-like 1.
(DOCX)
