# Supplementary figures and images for: Associations among circulating levels of follistatin-like 1, clinical parameters, and cardiovascular events in patients undergoing elective percutaneous coronary intervention with drug-eluting stents
Source: PLoS One. 2019 Apr 29;14(4):e0216297. doi: 10.1371/journal.pone.0216297 (PMC6488088; doi:10.1371/journal.pone.0216297)

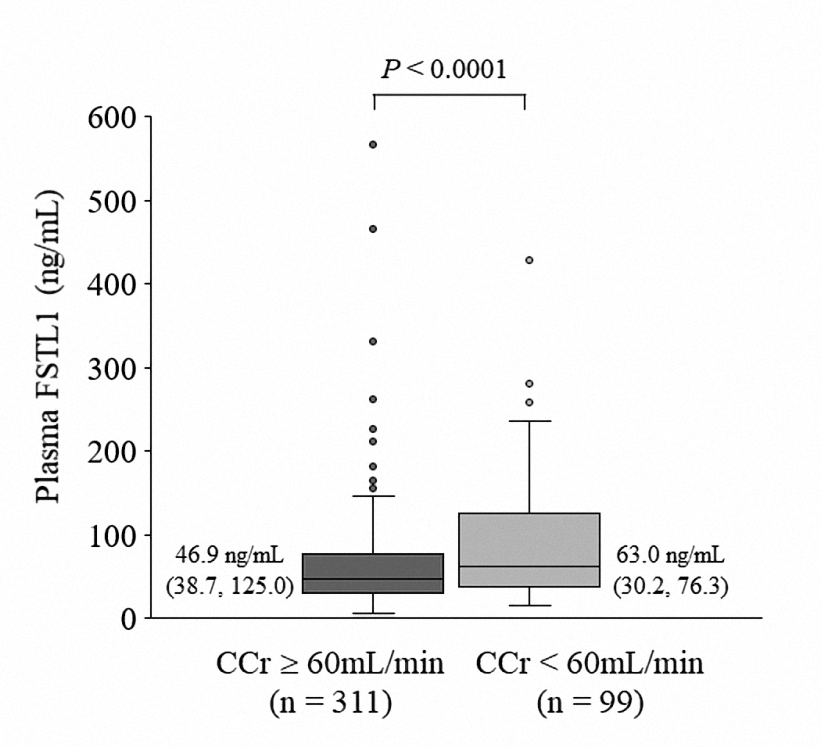

Supplement: S1 Fig — Patients with CCr < 60 mL/min had higher mean plasma FSTL1 than those with CCr ≥ 60 mL/min. FSTL1 levels were expressed as medians with the 25th and 75th percentiles. FSTL1, follistatin-like 1; CCr: creatinine clearance. (TIF) [file pone.0216297.s001.TIF]

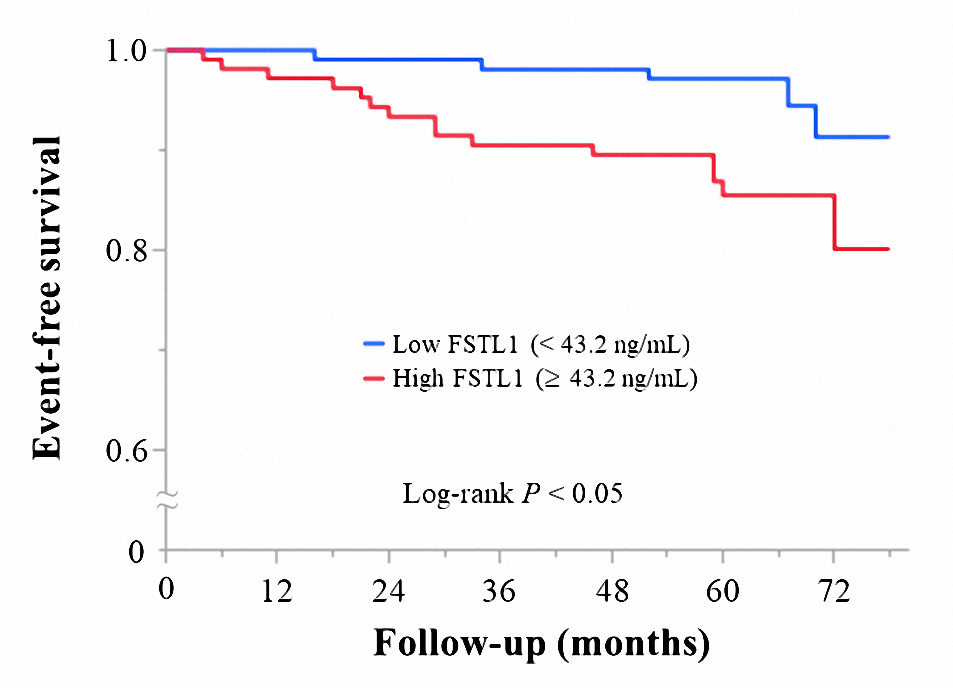

Supplement: S2 Fig — Kaplan–Meier analysis revealed that the MACCE rate was significantly higher in patients with FSTL1 (≥ 43.2 ng/mL) than in those FSTL1 < 43.2 ng/mL (P < 0.05). FSTL1, follistatin-like 1; MACCE, major adverse cardiac or cerebrovascular events. (TIF) [file pone.0216297.s002.TIF]

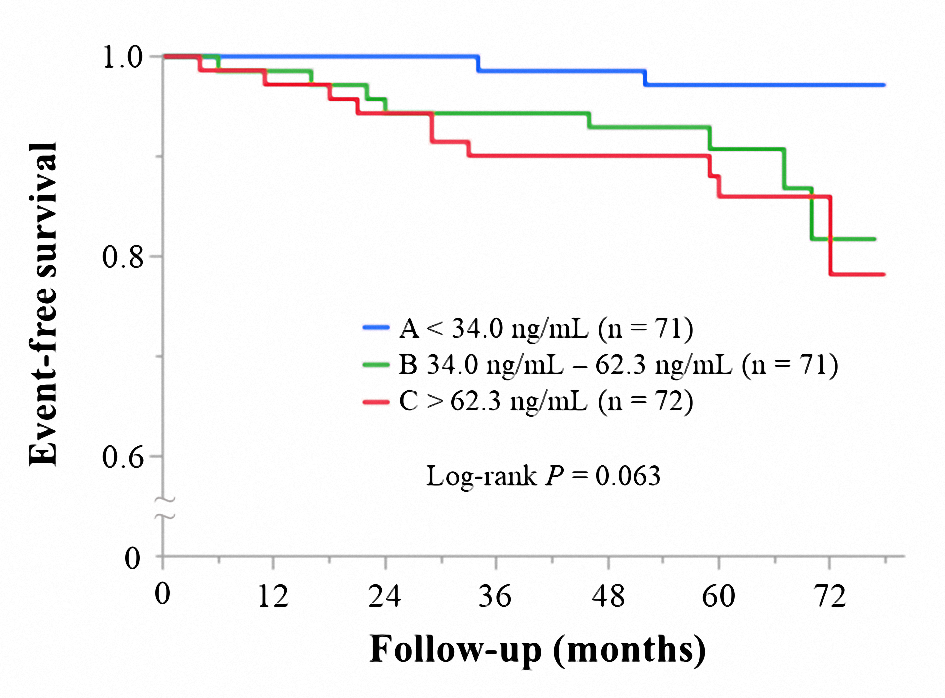

Supplement: S3 Fig — Kaplan–Meier analysis showed a trend but not a significant difference was noted among the three groups based on the tertiles FSTL1 levels. FSTL1, follistatin-like 1; MACCE, major adverse cardiac or cerebrovascular events. (TIF) [file pone.0216297.s003.TIF]

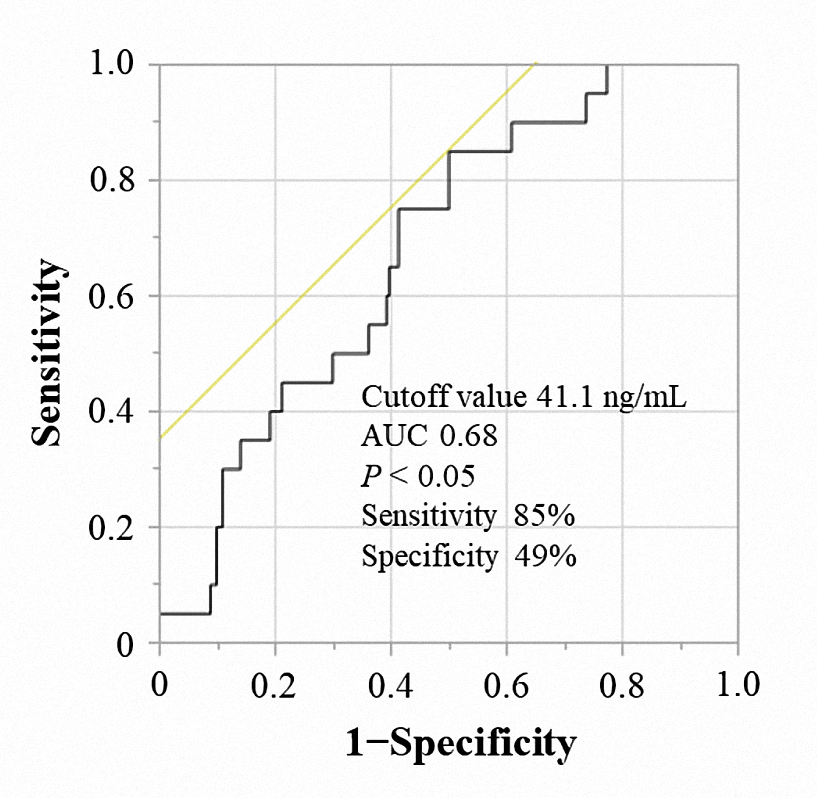

Supplement: S4 Fig — Receiver operating characteristics curve analysis estimated that an FSTL1 concentration of 41.1 ng/mL (area under the curve 0.68) had a sensitivity of 85% and specificity 49% for predicting MACCE (P < 0.05). FSTL1, follistatin-like 1; MACCE, major adverse cardiac or cerebrovascular events. (TIF) [file pone.0216297.s004.tif]
