# Supplementary material for: Associations among circulating levels of follistatin-like 1, clinical parameters, and cardiovascular events in patients undergoing elective percutaneous coronary intervention with drug-eluting stents
Source: PLoS One. 2019 Apr 29;14(4):e0216297. doi: 10.1371/journal.pone.0216297 (PMC6488088; doi:10.1371/journal.pone.0216297)
Supplement: S1 Table — MACCE, major adverse cardiac or cerebrovascular events; HR, hazard ratio; CI, confidence interval; NT-proBNP, N terminal pro brain natriuretic peptide; FSTL-1, follistatin-like 1. (DOCX) [file pone.0216297.s005.docx]

**S1 Table. Multivariate Cox Proportional Hazard Models for MACCE.**

|  | **Univariate** | | |  | **Multivariate** | | |
| --- | --- | --- | --- | --- | --- | --- | --- |
|  | **HR** | **95% CI** | ***P*** |  | **HR** | **95% CI** | ***P*** |
| Age, 1year increase | 0.96 | 0.91–1.02 | 0.16 |  | 0.97 | 0.91–1.03 | 0.28 |
| Gender (Male) | 0.50 | 0.18–1.75 | 0.24 |  | 0.33 | 0.098–1.33 | 0.11 |
| Body mass index, 1kg/m^2^ increase | 1.10 | 0.94–1.25 | 0.20 |  | 1.06 | 0.90–1.23 | 0.46 |
| Triglyceride, 1 mg/dL increase | 1.01 | 1.00–1.01 | <0.05 |  | 1.01 | 0.99–1.01 | 0.068 |
| Hemoglobin A1c, 1% increase | 1.28 | 1.00–1.57 | <0.05 |  | 1.06 | 0.77–1.42 | 0.72 |
| NT-pro BNP, 1 pg/mL increase | 1.001 | 1.000–1.002 | <0.01 |  | 1.001 | 0.999–1.002 | 0.47 |
| Diuretic usage | 6.90 | 2.44–17.22 | <0.001 |  | 2.82 | 0.45–9.71 | 0.25 |
| FSTL1 ≥ 43.2 ng/mL | 3.15 | 1.22–9.69 | <0.05 |  | 2.75 | 1.01–8.71 | <0.05 |

MACCE, major adverse cardiac or cerebrovascular events; HR, hazard ratio; CI, confidence interval; NT-proBNP, N terminal pro brain natriuretic peptide; FSTL-1, follistatin-like 1.
